# Supplementary figures and images for: Deep sampling and pooled amplicon sequencing reveals hidden genic variation in heterogeneous rye accessions
Source: BMC Genomics. 2020 Nov 30;21:845. doi: 10.1186/s12864-020-07240-3 (PMC7706248; doi:10.1186/s12864-020-07240-3)

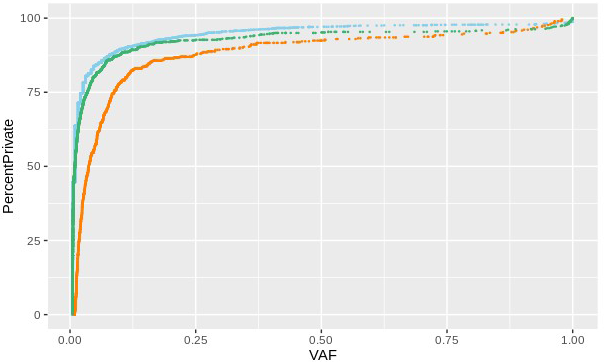

Supplement: Supplementary file 2 — Additional file 2: Figure S1. Percentage of private alleles (found in only one of the tested accessions) plotted by variant allele frequency (VAF). Data from GATK is plotted in light blue, CRISP in green and SNVer in orange. [file 12864_2020_7240_MOESM2_ESM.tif]

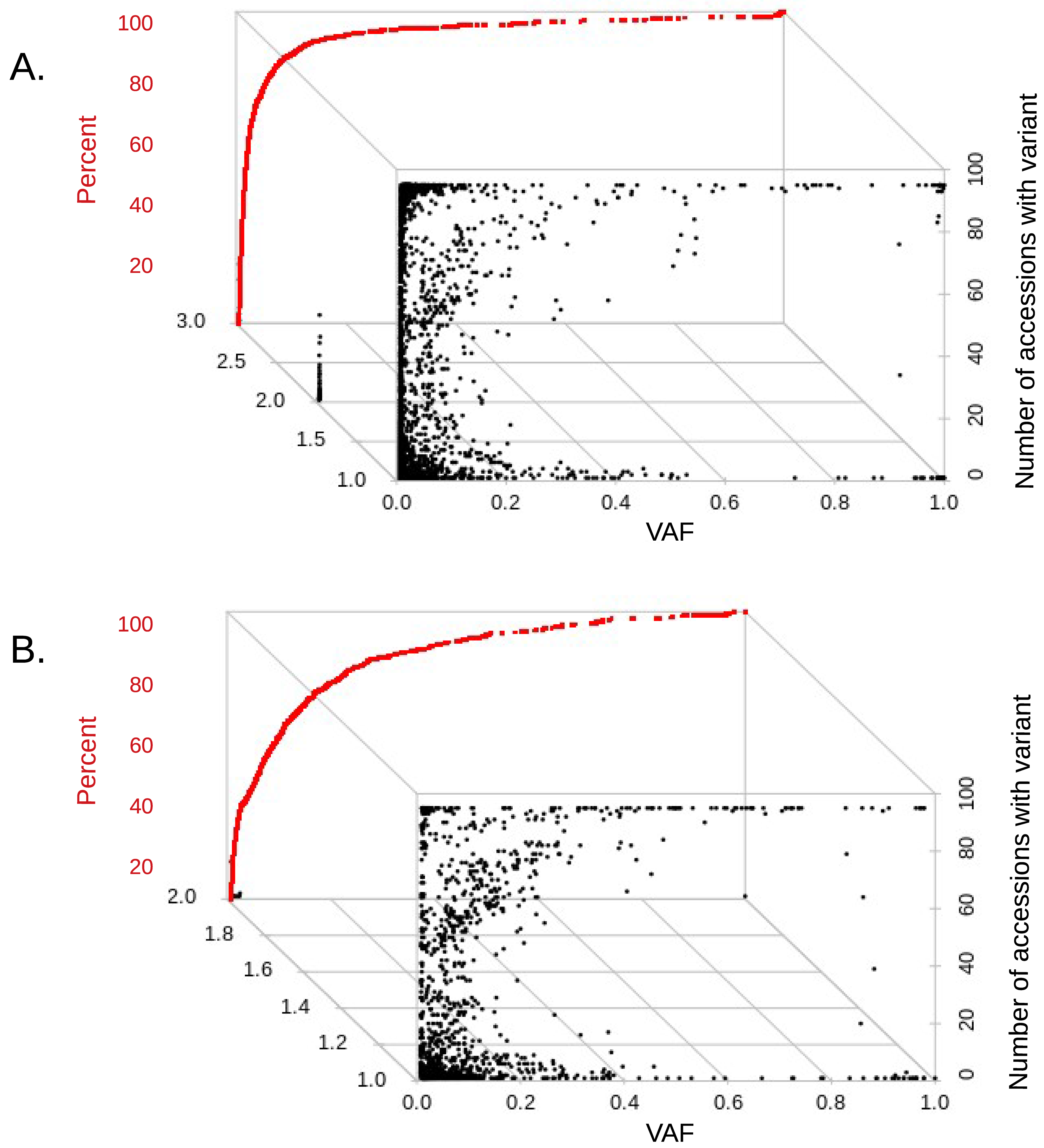

Supplement: Supplementary file 3 — Additional file 3: Figure S2. Scatter plots of variant allele frequency (VAF) data. VAF is plotted on the x-axis. Black dots represent every predicted variant. The number of accessions predicted to harbor the variant is plotted on the y-axis. Data is plotted on the z-axis to separate different variants that share the same VAF and number of accessions. The percentage of the total data from VAF 0 to a specific frequency is overlaid in red. Variants predicted by CRISP are plotted in panel A, and by SNVer in panel B. [file 12864_2020_7240_MOESM3_ESM.tif]

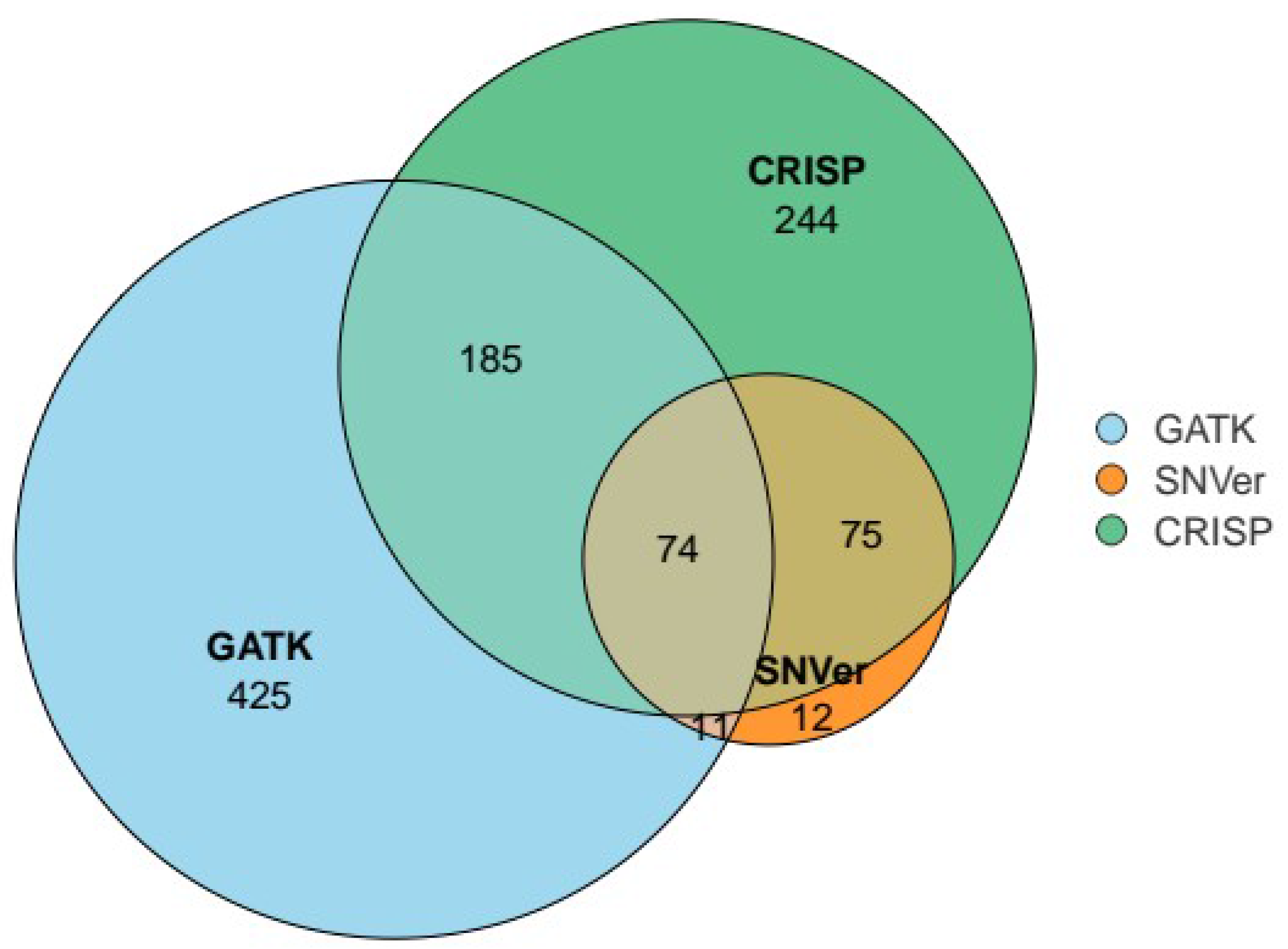

Supplement: Supplementary file 5 — Additional file 5: Figure S3. Venn diagram of variants called by GATK, SNVer and CRISP predicted to be deleterious using SIFT. [file 12864_2020_7240_MOESM5_ESM.tif]

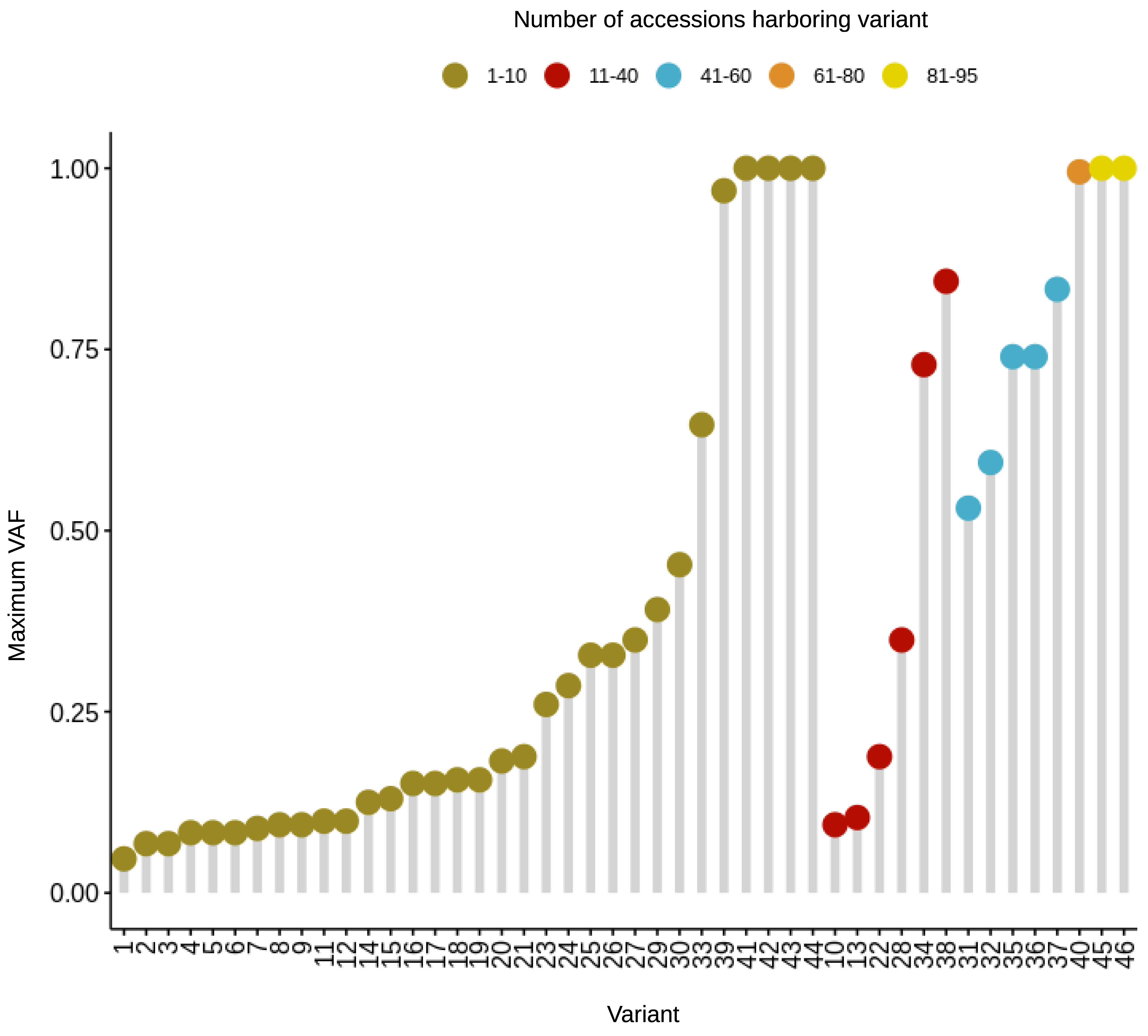

Supplement: Supplementary file 6 — Additional file 6: Figure S4. Lollipop chart of allele frequencies of GATK variants predicted deleterious by SIFT and also called by SNVer and CRISP. Each variant is assigned an arbitrary number (x axis) with maximum allele frequency values calculated from GATK VCF data is plotted on the y axis. Data is sorted into 5 distinct groups based on the number of accessions harboring the variant. This sorting is indicated by the colored ball at the end of the bar. Allele frequencies below 0.039 are not plotted. [file 12864_2020_7240_MOESM6_ESM.tif]

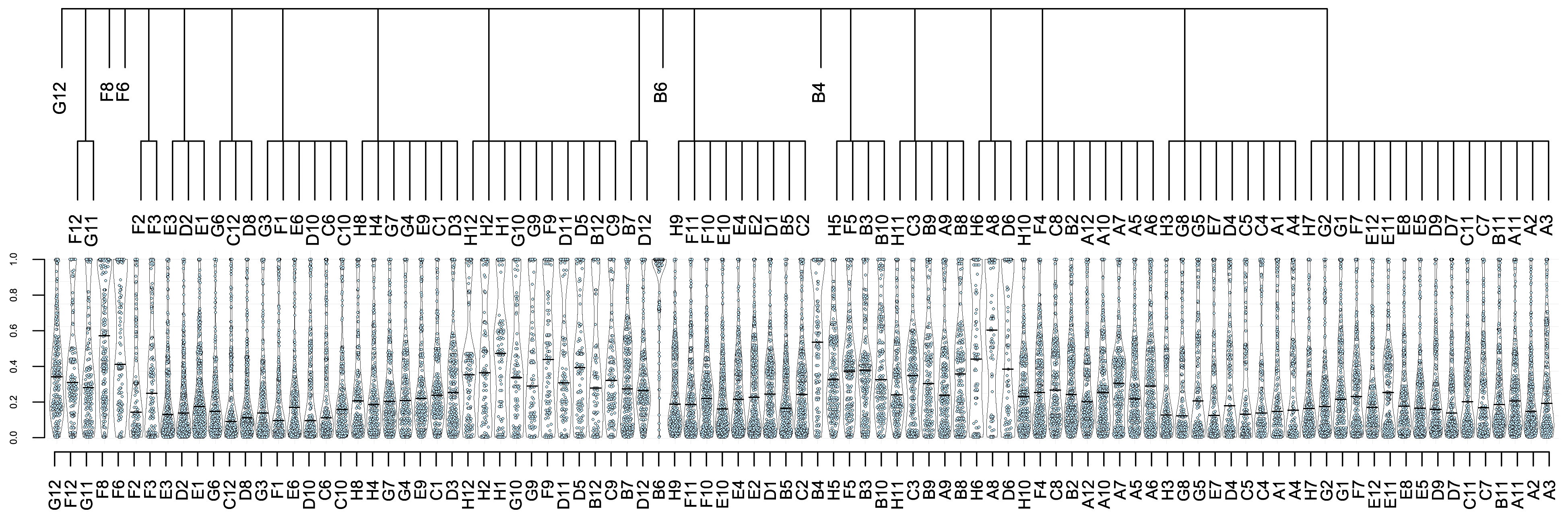

Supplement: Supplementary file 10 — Additional file 10: Figure S6. Dendrogram showing relationships between distributions of VAF values (shown as strip/violin plots) for 95 accessions (based on results of the two-part Wilcoxon test). Horizontal bars indicate median values. [file 12864_2020_7240_MOESM10_ESM.tif]

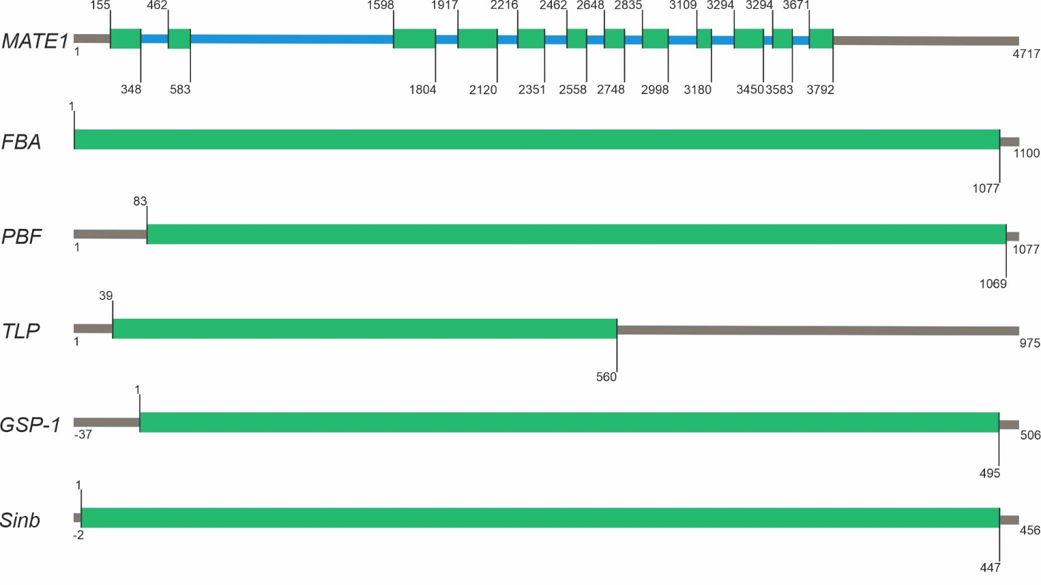

Supplement: Supplementary file 13 — Additional file 13: Figure S7. Target regions used in this study. Introns are colored blue, non-coding sequence grey and exons green. Relative nucleotide positions in base pairs are listed. [file 12864_2020_7240_MOESM13_ESM.jpg]
